# Supplementary material for: Dynamics of SARS-CoV-2 IgG in Nursing Home Residents in Belgium Throughout Three BNT162b2 Vaccination Rounds: 19-Month Follow-Up
Source: Vaccines (Basel). 2025 Apr 15;13(4):409. doi: 10.3390/vaccines13040409 (PMC12030799; doi:10.3390/vaccines13040409)
Supplement: Supplementary file 1 [file vaccines-13-00409-s001.zip › vaccines-3553726-supplementary.pdf]

Supplementary File

SUBSET I: In the SCOPE2 study, DBS were immediately analyzed for S1RBD IgG after collection and therefore S1RBD IgG levels were already available for these samples. In the SCOPE1 study, DBS were collected in addition to SARS-CoV-2 antibody rapid tests, but only subsets of DBS were previously analyzed for previously published work [4]. To have a complete 19-month overview of S1RBD IgG levels over time in NHR, we selected a subset by random sampling of 200 NHR that were included in SCOPE2, across the 30 NH included in SCOPE2 (subset I). For this subset, the samples from SCOPE1 were retrospectively analyzed for S1RBD IgG quantity.

SUBSET II: Within the SCOPE1 study, non-responders were identified. Non-responders were defined when a subject's first and/or second following SARS-CoV-2 antibody rapid test result ≥14 days after primary course vaccination was negative. From all non-responders, 61 subjects were selected with an available DBS sample for the timepoint ≥ 14 days after vaccination. For comparison, a responder group was randomly selected (n=60) from the remainder of subjects in the SCOPE1 study matched for participant type, infection history and vaccination status of non-responders in a 1:1 ratio. SARS-CoV-2 breakthrough cases were excluded from the analysis after the timepoint of infection (n=23; 14 among non-responders, 9 among responders). For both the responders and non-responders, DBS were analyzed for the four timepoints after primary course vaccination, and one time point after booster vaccination.

SUBSET III: All NHR included in the SCOPE2 study (n=492) were divided into two groups, those that received a second booster during follow-up (n=255) and those who did not (n=237). For the group that received a second booster during follow-up, 69 subjects were further excluded as they received a second booster before sampling in June 2022 and 5 were excluded because of a breakthrough infection between June and September 2022. For the group that did not receive a second booster during follow-up, 7 subjects were excluded because of a breakthrough infection between June and September 2022. Therefore, in total, 411 subjects were included in this analysis.

Supplementary Table S1. Between and within-subject standard deviations and variances, and missing data per model. n=number.

Model	Subject	Standard Deviation	Variance	Missing observations/total observations n, %
Infection-naive versus infection-primed	Between subject	0.5918	0.3502	512/1600, 32%
	Within subject	0.4307	0.1855	
Non-responders	Between subject	0.4037	0.1630	45/605, 7%
	Within subject	0.3979	0.1583	
Second booster	Between subject	0.4619	0.2134	44/822, 5%
	Within subject	0.3177	0.1009	

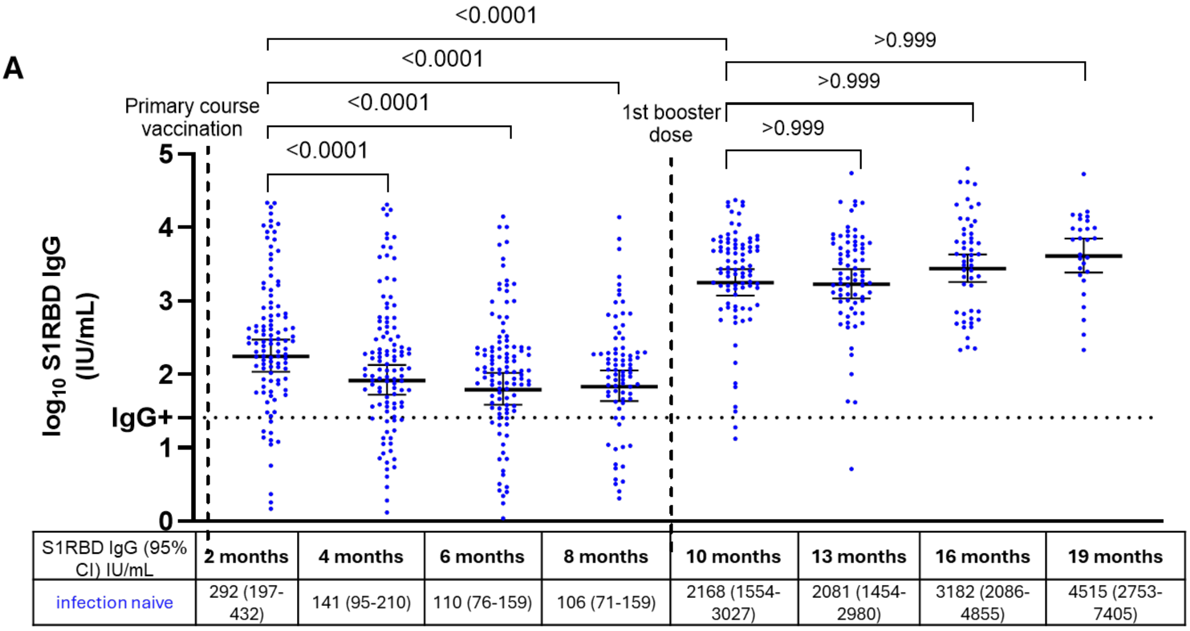

S1RBD IgG (95% CI) IU/mL	2 months	4 months	6 months	8 months	10 months	13 months	16 months	19 months
infection naive	292 (197-432)	141 (95-210)	110 (76-159)	106 (71-159)	2168 (1554-3027)	2081 (1454-2980)	3182 (2086-4855)	4515 (2753-7405)

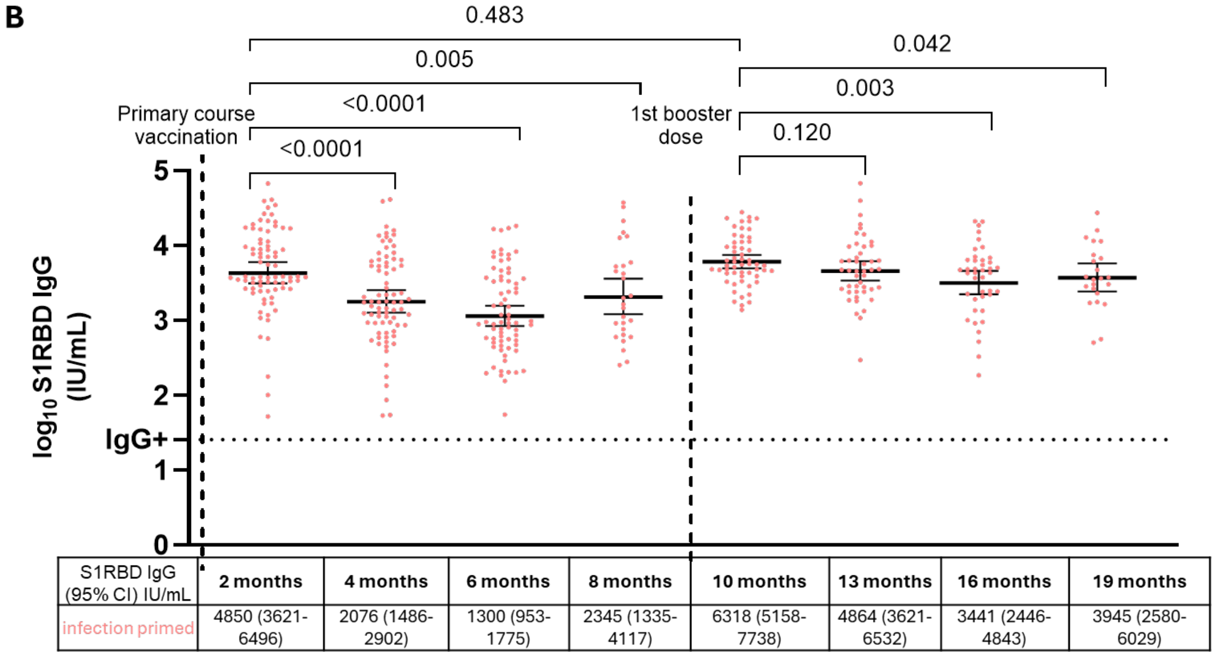

S1RBD IgG (95% CI) IU/mL	2 months	4 months	6 months	8 months	10 months	13 months	16 months	19 months
infection primed	4850 (3621-6496)	2076 (1486-2902)	1300 (953-1775)	2345 (1335-4117)	6318 (5158-7738)	4864 (3621-6532)	3441 (2446-4843)	3945 (2580-6029)

Supplementary Figure S1. S1RBD IgG dynamics in infection-naive (A) (n=119) and infection-primed NHR (B) (n=81) after primary course vaccination and first booster administration. The upper graph (A) represents infection-naive NHR and the lower graph (B) represents infection-primed NHR. S1RBD IgG geometric mean concentrations (GMC) in IU/mL with 95% CI are presented per timepoint below the graph and log10 GMCs by horizontal lines with error bars. The first timepoint after vaccination was considered as baseline. NHR

with a breakthrough infection and those who received a second booster were excluded at that respective timepoint. The horizontal dashed line represents the cutoff for SARS-CoV-2 seropositivity (log10 26 IU/mL). IU/mL: international units/mL.CI: Confidence Interval, ns: not significant.
